# Supplementary material for: Determining Aspergillus fumigatus transcription factor expression and function during invasion of the mammalian lung
Source: PLoS Pathog. 2021 Mar 29;17(3):e1009235. doi: 10.1371/journal.ppat.1009235 (PMC8031882; doi:10.1371/journal.ppat.1009235)
Supplement: S1 Fig — Shown are NanoString probe counts for 97 environmentally responsive A. fumigatus genes in lung samples from 3 mice after 5 d of infection and a single immunosuppressed, uninfected mouse. (PDF) [file ppat.1009235.s001.pdf]

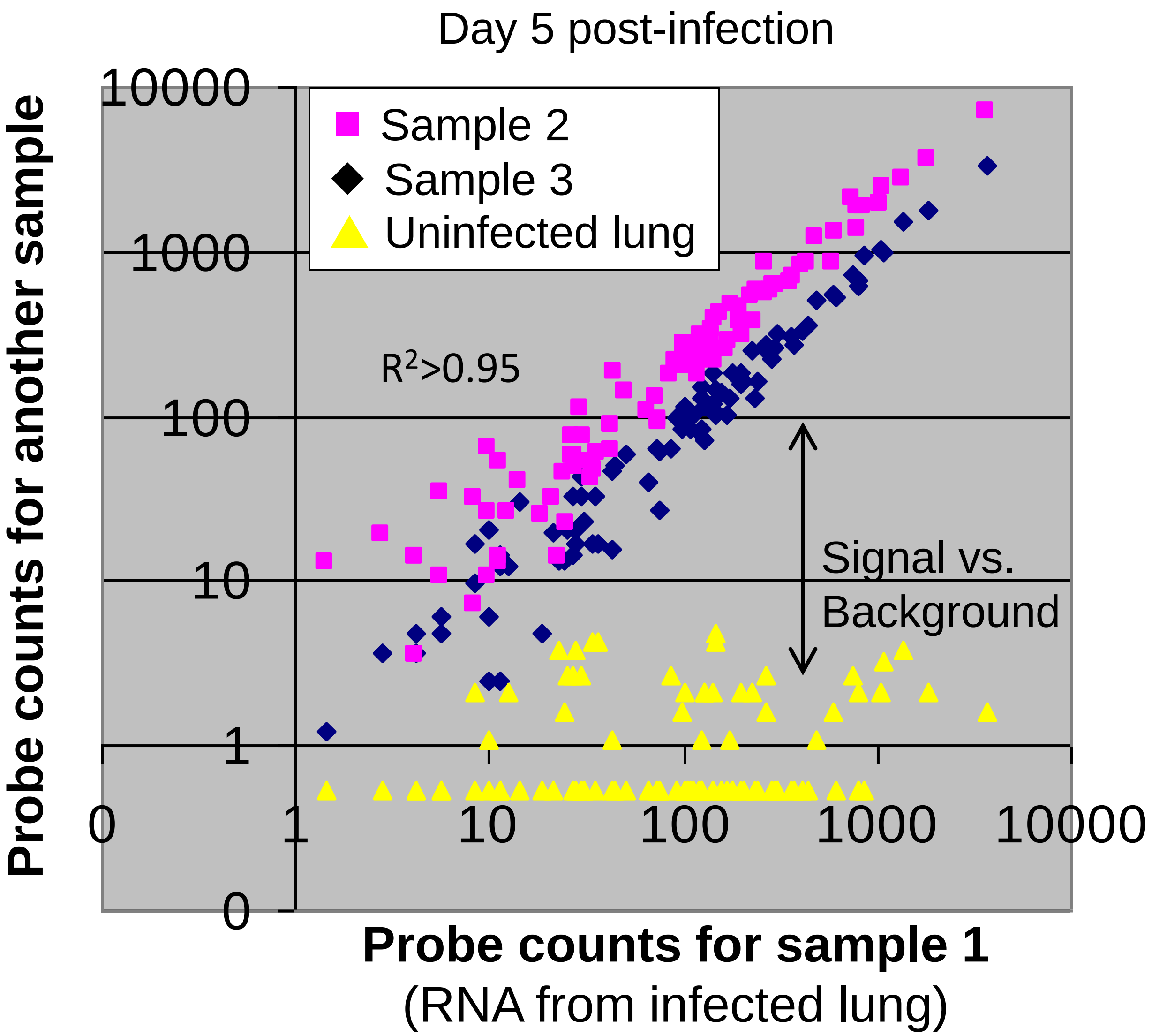

S1 Fig. High reproducibility and low background of *in vivo* NanoString data. Shown are NanoString probe counts for 97 environmentally responsive *A. fumigatus* genes in lung samples from 3 mice after 5 d of infection and a single immunosuppressed, uninfected mouse.
