# Supplementary material for: Determining Aspergillus fumigatus transcription factor expression and function during invasion of the mammalian lung
Source: PLoS Pathog. 2021 Mar 29;17(3):e1009235. doi: 10.1371/journal.ppat.1009235 (PMC8031882; doi:10.1371/journal.ppat.1009235)
Supplement: S2 Fig — Shown are the lung masses of mice after 5 d of infection with the indicated strains of A. fumigatus. Lines indicate the median and interquartile ranges of 5 mice per strain. Symbols indicate the results of individual mice. *P < 0.05. (PDF) [file ppat.1009235.s002.pdf]

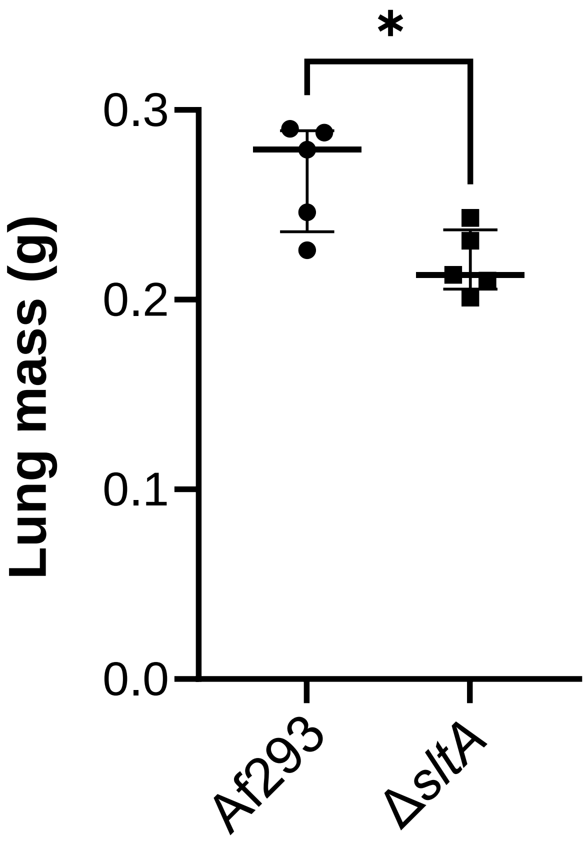

S2 Fig. Mice infected with the Δ*sltA* mutant have lower lung mass. Shown are the lung masses of mice after 5 d of infection with the indicated strains of *A. fumigatus.* Lines indicate the median and interquartile ranges of 5 mice per strain. Symbols indicate the results of individual mice. *$P < 0.05$.
