# Supplementary material for: Determining Aspergillus fumigatus transcription factor expression and function during invasion of the mammalian lung
Source: PLoS Pathog. 2021 Mar 29;17(3):e1009235. doi: 10.1371/journal.ppat.1009235 (PMC8031882; doi:10.1371/journal.ppat.1009235)
Supplement: S3 Fig — Serial 10-fold dilutions of the indicated strains of A. fumigatus were spotted onto Aspergillus minimal medium (AMM) containing the indicated stressors. The plates were imaged after incubation at 37°C for 2 d. (PDF) [file ppat.1009235.s003.pdf]

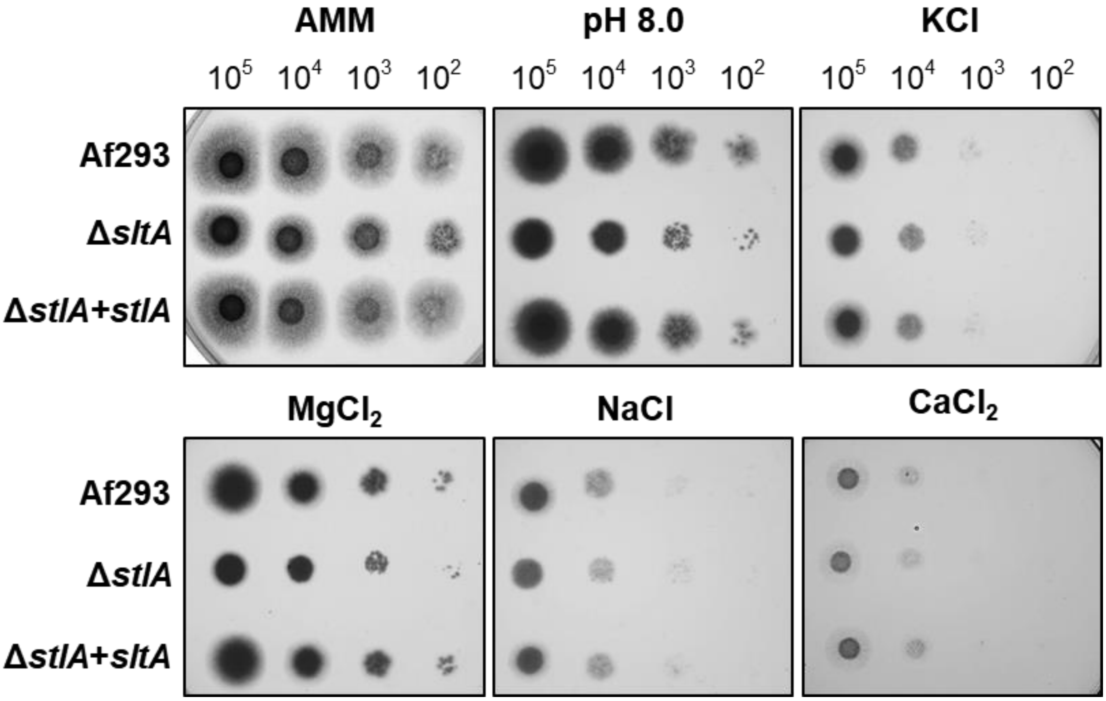

S3 Fig. The Δ*sltA* mutant has wild-type growth at alkaline pH and in the presence of cations. Serial 10-fold dilutions of the indicated strains of *A. fumigatus* were spotted onto *Aspergillus* minimal medium (AMM) containing the indicated stressors. The plates were imaged after incubation at 37°C for 2 d.
