# Supplementary material for: Determining Aspergillus fumigatus transcription factor expression and function during invasion of the mammalian lung
Source: PLoS Pathog. 2021 Mar 29;17(3):e1009235. doi: 10.1371/journal.ppat.1009235 (PMC8031882; doi:10.1371/journal.ppat.1009235)
Supplement: S4 Fig — Values represent Log2 expression ratios for each gene in the ΔsltA strain divided by the wild-type strain as determined by RNA-seq (Af293) or qRT-PCR (AF1160). (PPTX) [file ppat.1009235.s004.pptx]

## Slide 1
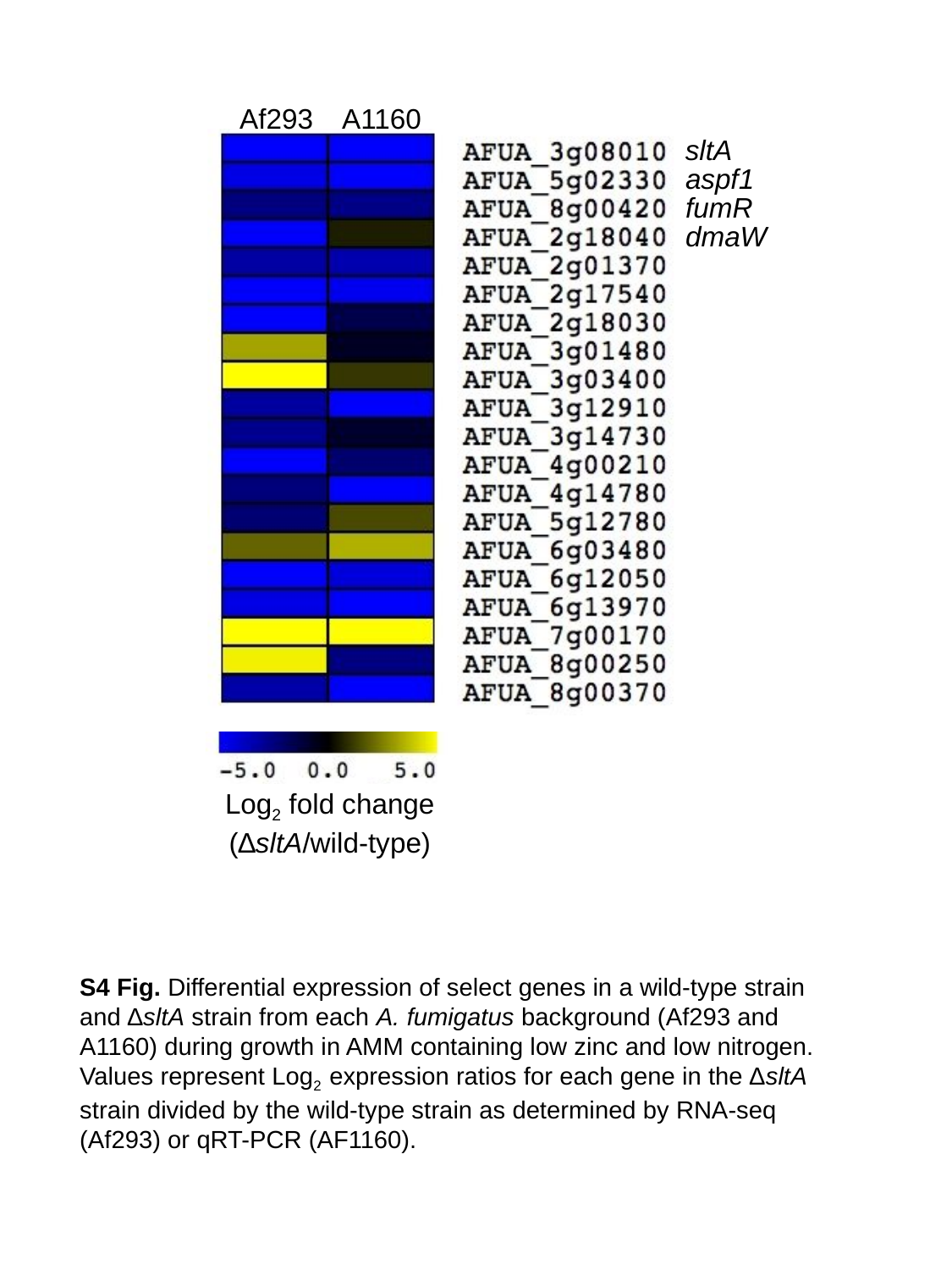

Af293
A1160
sltA
aspf1
fumR
dmaW
Log2 fold change
(∆sltA/wild-type)
S4 Fig. Differential expression of select genes in a wild-type strain and ∆sltA strain from each A. fumigatus background (Af293 and A1160) during growth in AMM containing low zinc and low nitrogen. Values represent Log2 expression ratios for each gene in the ∆sltA strain divided by the wild-type strain as determined by RNA-seq (Af293) or qRT-PCR (AF1160).
