# Supplementary material for: Determining Aspergillus fumigatus transcription factor expression and function during invasion of the mammalian lung
Source: PLoS Pathog. 2021 Mar 29;17(3):e1009235. doi: 10.1371/journal.ppat.1009235 (PMC8031882; doi:10.1371/journal.ppat.1009235)
Supplement: S5 Fig — (A) qRT-PCR results of relative gene expression in the indicated strains. (B) The replacement of nscR (Afu7g00130) by the hygromycin resistance cassette (HYG) was verified by PCR using two different sets of primers. (PDF) [file ppat.1009235.s005.pdf]

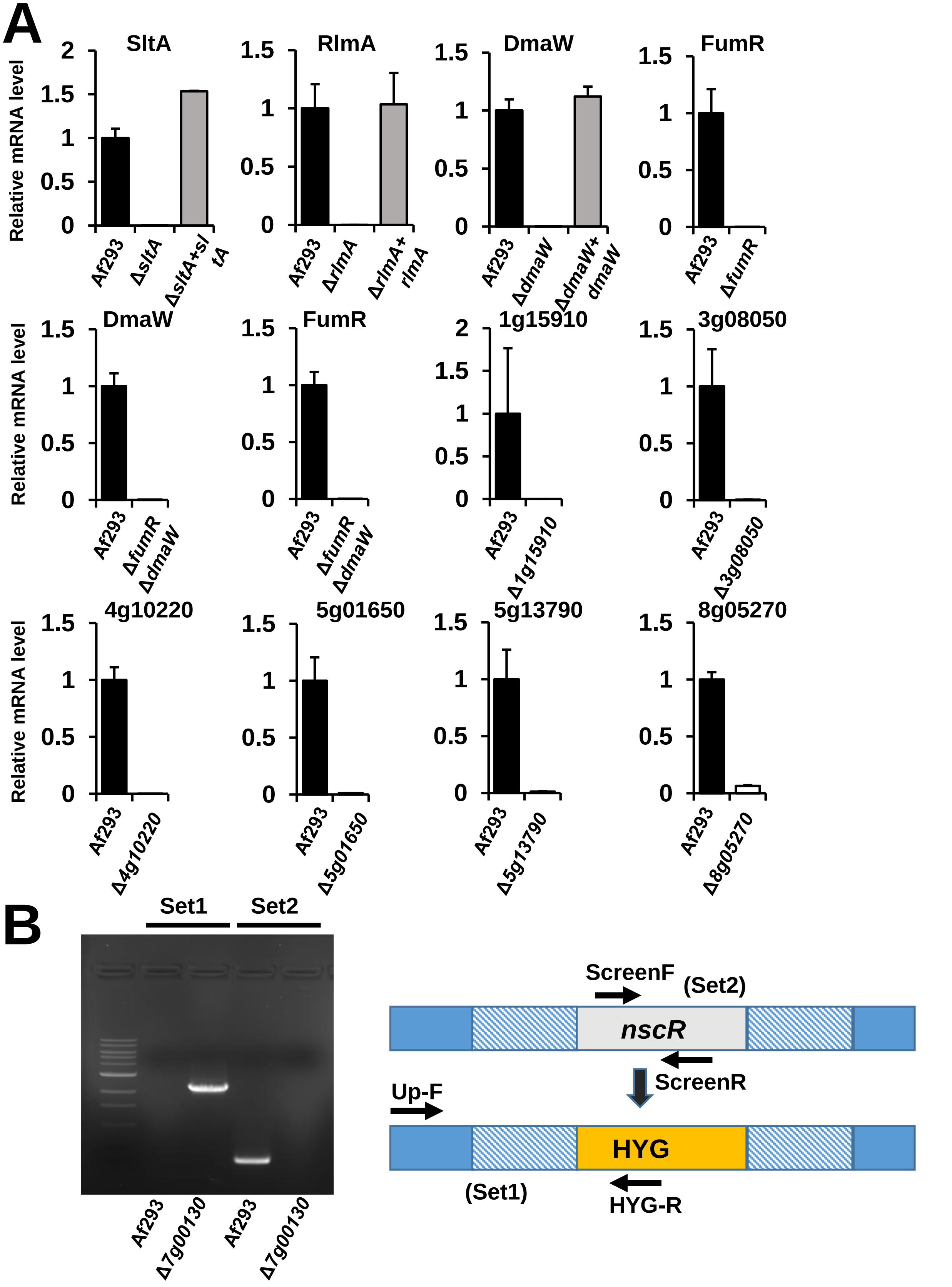

S5 Fig. Verification of gene deletion in the strains construted in current study. (A) qRT-PCR results of relative gene expression in the indicated strains. (B) The replacement of *nscR* (Afu7g00130) by the hygromycin resistance cassette (HYG) was verified by PCR using two different sets of primers.
